# Unveiling the role of Mfsd2a and LPC-DHA in kidney repair

**DOI:** 10.1016/j.jlr.2023.100422

**Published:** 2023-08-06

**Authors:** Rosalie G.J. Rietjens, Ton J. Rabelink

**Affiliations:** 1 Department of Internal Medicine (Nephrology) & Einthoven Laboratory of Vascular and Regenerative Medicine, Leiden University Medical Center, Leiden, The Netherlands; 2 The Novo Nordisk Foundation Center for Stem Cell Medicine (reNEW), Leiden University Medical Center, Leiden, The Netherlands

Chronic kidney disease and its subsequent progression to kidney failure has become a growing global health concern, making it one of the leading causes of disease burden worldwide (1). When the kidneys face insults such as surgery or viral infections, the restorative capacity of renal epithelial cells, particularly in the proximal tubules, is put to the test. Acute-on-chronic kidney disease often leads to the failure of epithelial dedifferentiation and redifferentiation, resulting in kidney fibrosis and the eventual need for renal replacement therapy (Fig. 1) (2). Therefore, gaining a comprehensive understanding of the repair processes involved in kidney injury is crucial for maintaining kidney health.

**Fig. 1:**
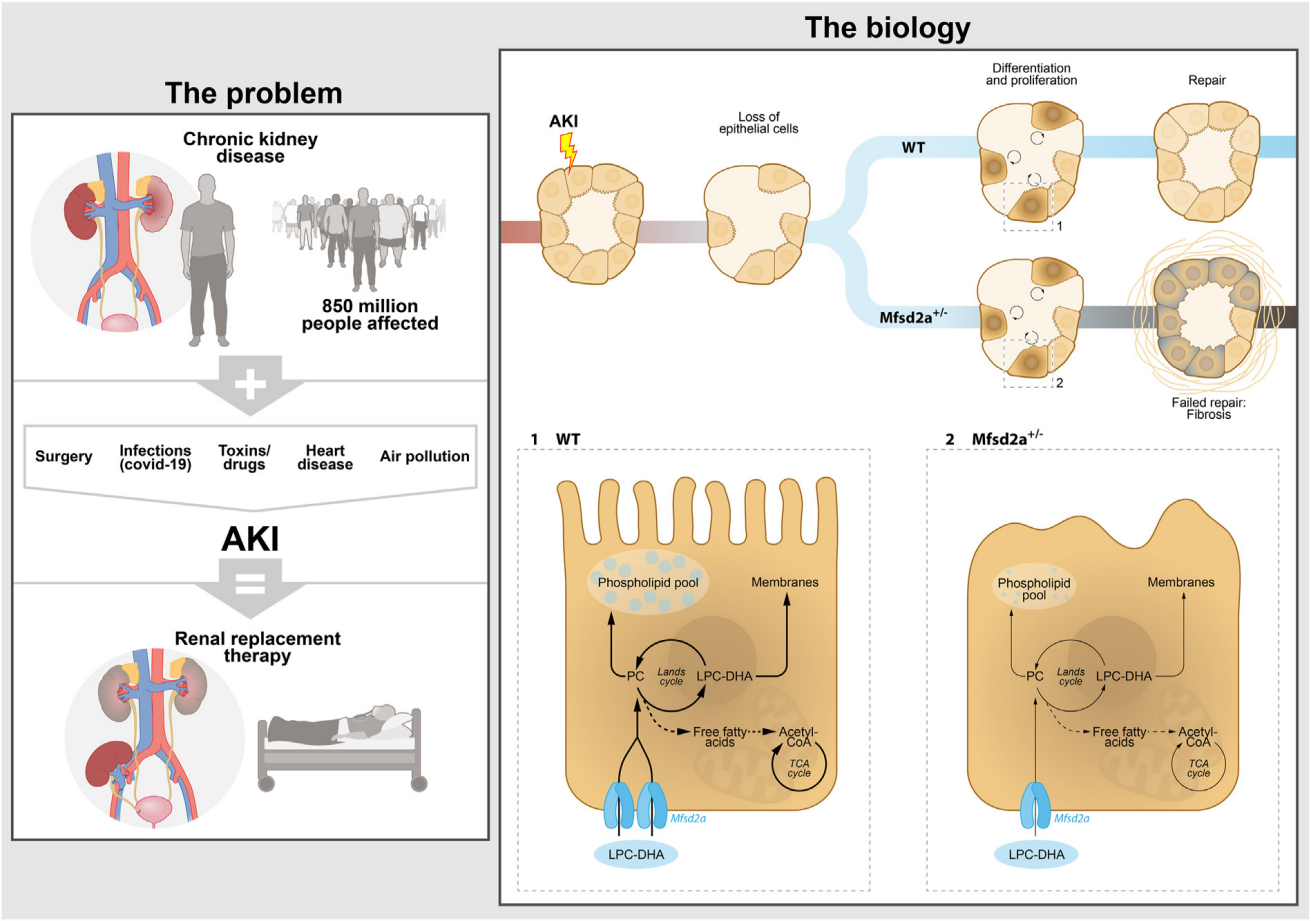
The role of Mfsd2a in proximal tubule repair upon acute-on-chronic kidney disease. The problem: The burden of renal replacement therapy needed after acute-on-chronic kidney disease is a global problem affecting millions of people. The biology: Loke *et al.* identified Mfsd2a as crucial transporter during epithelial repair. Uptake of blood-derived LPC-DHA through Mfsd2a allows energy-conservative replenishment of the phospholipid pool through the Lands’ cycle. Furthermore, it provides a building block for cellular membranes and the epithelial brush border. Free fatty acids released in the cycle might aid in fueling the tricarboxylic acid cycle, restoring the metabolic balance in the injured proximal tubular cells.

Recent studies have shed light on the role of metabolic dysregulation in kidney repair. Metabolic processes, such as the tricarboxylic acid cycle and lipid remodeling, have emerged as critical factors in determining cell fate decisions during injury and repair (3). Metabolic alterations, including changes in phospholipid composition, have been identified as early hallmarks of kidney damage associated with conditions like diabetes (4, 5, 6). In this issue of the *Journal of Lipid Research*, Loke *et al.* explored the pivotal role of lipid metabolism and remodeling in kidney repair following acute kidney injury (AKI).

The study focused on investigating the function of the Mfsd2a transporter in the kidney. Previously known for its role in the blood-brain barrier and blood-retinal barrier, Mfsd2a acts as the primary transporter for lysophosphatidylcholine (LPC) uptake, with a preference for the omega-3 fatty acid DHA (7). Disruption of Mfsd2a has been linked to abnormal brain development and neurological disorders (8, 9, 10). However, its function in other tissues, particularly in healthy and disease states, remained unclear. Loke *et al.* discovered that Mfsd2a was exclusively expressed in the S3 segment of the renal proximal tubule, which is highly susceptible to AKI. By employing an activity probe called LPC-LightOx, the researchers confirmed that Mfsd2a facilitated the transport of blood-derived LPC from the basolateral membranes of the S3 segment into the renal epithelium. To elucidate the role of Mfsd2a in kidney repair, the researchers exposed both WT and Mfsd2a haploinsufficient (HET) mice to ischemia-reperfusion injury (IRI) to induce AKI. While both groups initially experienced similar levels of kidney failure, WT mice demonstrated prompt recovery of kidney function in the days following IRI, whereas HET mice displayed impaired urinary concentration ability and severe renal injury at day 10 post-IRI. These findings underscored the critical role of Mfsd2a in kidney repair after AKI. The investigators then sought to uncover the underlying mechanism of Mfsd2a′s contribution to kidney repair. Membrane integrity and composition are vital for cellular functionality, and cellular differentiation and proliferation necessitate rapid replenishment of the membrane lipid pool. Considering the role of Mfsd2a as a transporter of an essential phospholipid involved in cellular membranes, the authors hypothesized that Mfsd2a contributes to phospholipid homeostasis during the recovery process. Lipidomic analysis revealed alterations in lipid composition in HET mice at day 10 post-IRI, particularly a reduction in the total DHA pool and DHA-containing phospholipids. This deficiency in DHA may explain the impaired kidney repair observed in Mfsd2a haploinsufficiency. To validate their hypothesis, the researchers supplemented HET mice with LPC-DHA after injury induction, resulting in significant improvements in renal function, histopathological markers of kidney injury, and reversal of lipidomic changes.

The study by Loke *et al.* unveils a critical mechanism whereby phospholipid transport by Mfsd2a into renal epithelial cells plays a crucial role in brush border recovery following AKI. Proper homeostasis of intracellular phospholipids, facilitated by Mfsd2a or LPC-DHA supplementation, enables the S3 epithelium to restore its brush border and supports epithelial proliferation. In addition, the study highlights the metabolic aspects of kidney repair. The high metabolic demand of the proximal tubule S3 segment, coupled with the ATP depletion caused by IRI, necessitates an energy-conserving cellular repair strategy. Phospholipids not only rebuild the epithelial brush border but also provide the building blocks for the proliferation of new epithelial cells (11). The remodeling of phospholipids through the Lands' cycle may also fuel the tricarboxylic acid cycle, contributing to the metabolic regulation of recovering proximal tubules (Fig. 1).

The newly uncovered role of Mfsd2a and LPC-DHA in renal recovery holds significant potential for clinical implications. Considering that acute-on-chronic disease and subsequent tubular failure are major contributors to the need for renal replacement therapy, LPC-DHA supplementation following an acute event, such as surgery or viral infection, could enhance the intrinsic restorative capacity of the vulnerable proximal tubule S3 segment. Moreover, LPC-DHA supplementation could prove beneficial in kidney transplantation settings. Pretransplantation supplementation of the donor kidney with LPC-DHA during organ perfusion could potentially improve the function of the donor organ, leading to improved transplantation outcomes.

In conclusion, this study provides novel insights into the crucial role of blood-derived phospholipid uptake by the proximal tubule S3 segment in renal recovery following AKI. The energy-efficient manner of membrane replenishment in the metabolically challenged S3 segment, supporting redifferentiation and proliferation, offers a potential explanation for the indispensable role of Mfsd2a. These findings open new avenues for therapeutic strategies aimed at improving kidney repair and ultimately enhancing renal health.

## Conflict of interest

The authors declare that they have no conflicts of interest with the contents of this article.
